# Supplementary material for: Complex‐centric proteome profiling by SEC‐SWATH‐MS
Source: Mol Syst Biol. 2019 Jan 14;15(1):e8438. doi: 10.15252/msb.20188438 (PMC6346213; doi:10.15252/msb.20188438)
Supplement: Supplementary file 8 — Dataset EV7 [file MSB-15-e8438-s008.zip › feature_plots_string/Q6UXN9.pdf]

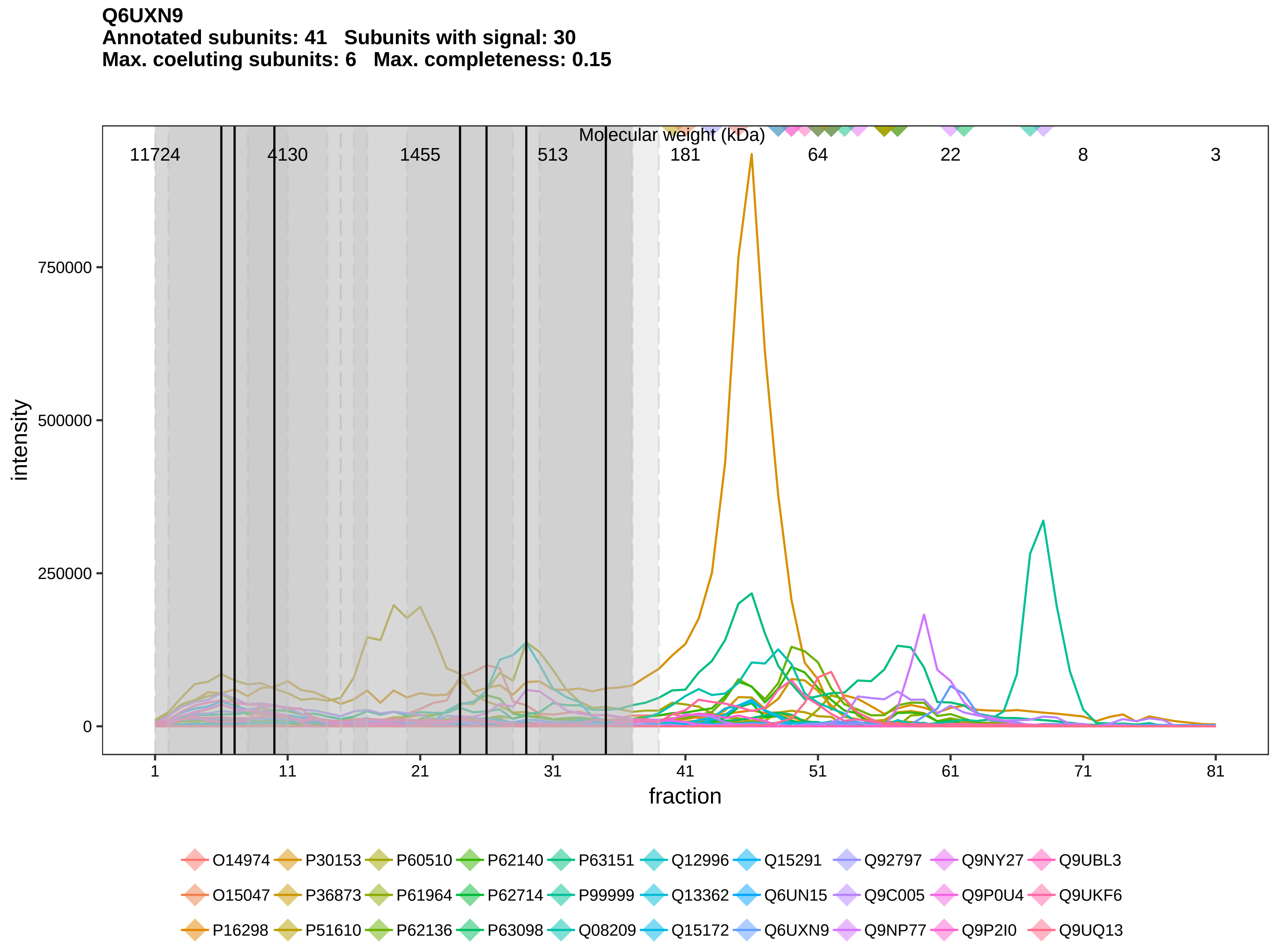

Q6UXN9
Annotated subunits: 41 Subunits with signal: 30
Max. coeluting subunits: 6 Max. completeness: 0.15
Molecular weight (kDa)
11724
4130
1455
513
181
64
22
8
3
intensity
750000
500000
250000
0
1
11
21
31
41
51
61
71
81
fraction
O14974
P30153
P60510
P62140
P63151
Q12996
Q15291
Q92797
Q9NY27
Q9UBL3
O15047
P36873
P61964
P62714
P99999
Q13362
Q6UN15
Q9C005
Q9P0U4
Q9UKF6
P16298
P51610
P62136
P63098
Q08209
Q15172
Q6UXN9
Q9NP77
Q9P2I0
Q9UQ13
